# Underweight and Mortality in Type 2 Diabetes: A Nationwide Retrospective Cohort Study

**DOI:** 10.1002/jcsm.70145

**Published:** 2025-12-05

**Authors:** Hun Jee Choe, Kyong Do Han, Ji‐Hong Park, Jiwoo Lee, Mi Kyung Kwak, Yun Mi Choi, Sun‐Joon Moon, Eun‐Gyoung Hong

**Affiliations:** ^1^ Division of Endocrinology and Metabolism, Department of Internal Medicine, Hallym University Dongtan Sacred Heart Hospital Hallym University School of Medicine Hwaseong Republic of Korea; ^2^ Department of Statistics and Actuarial Science Soongsil University Seoul Republic of Korea; ^3^ Division of Endocrinology and Metabolism, Department of Internal Medicine, Kangbuk Samsung Hospital Sungkyunkwan University School of Medicine Seoul Republic of Korea

**Keywords:** body mass index, cardiovascular mortality, cerebrovascular mortality, diabetes, mortality, underweight

## Abstract

**Background:**

Being underweight is an underrecognized risk factor for mortality among individuals with type 2 diabetes (T2D). This study aimed to evaluate the associations of underweight status with mortality among individuals with T2D.

**Methods:**

This nationwide, retrospective, population‐based cohort study used data from the Korean National Health Insurance Service. We included 1 788 996 adults with T2D who underwent baseline screening between 1 January 2015 and 31 December 2016, with follow‐up through December 31, 2022. Underweight was defined as body mass index (BMI) < 18.5 kg/m^2^ and further categorized as mild (17.0–18.4 kg/m^2^), moderate (16.0–16.9 kg/m^2^), and severe (< 160 kg/m^2^). The primary outcome was all‐cause mortality analysed using multivariable Cox proportional hazards regression.

**Results:**

During a median follow‐up of 6.96 years, 176 056 deaths occurred. Compared with the non‐underweight group (BMI ≥ 18.5 kg/m^2^), all‐cause mortality increased stepwise across underweight categories, with adjusted hazard ratios (aHRs) of 2.037 (95% CI, 1.982–2.094) for mild underweight, 2.719 (2.587–2.857) for moderate underweight, and 3.876 (3.646–4.119) for severe underweight. Cause‐specific mortality showed similar gradients. For diabetes‐related mortality, the aHRs were 2.265 (2.073–2.474), 3.306 (2.845–3.843) and 5.136 (4.300–6.134) across mild, moderate and severe underweight, respectively; for cardiovascular mortality, 1.881 (1.721–2.055), 2.087 (1.751–2.487) and 2.825 (2.267–3.519); and for cerebrovascular mortality, 2.100 (1.881–2.344), 2.300 (1.846–2.866) and 3.501 (2.702–4.537). Notably, the severe underweight group (BMI < 16.0 kg/m^2^) showed significantly higher all‐cause mortality than the severe obesity group (BMI ≥ 35.0 kg/m^2^) when compared to the BMI 25.0–29.9 kg/m^2^ reference group (aHR [95% CI]: 5.168 [4.857–5.499] vs. 1.504 [1.418–1.595]).

**Conclusions:**

Among individuals with T2D, underweight status was associated with substantially elevated mortality risks, with severe underweight exhibiting greater risk than severe obesity.

## 1 Introduction

Type 2 diabetes (T2D) and obesity, which share several common metabolic derangements, are closely intertwined [1]. Severe obesity is associated with increased mortality, particularly mortality attributable to cardiovascular and other metabolic complications [2]. Therefore, weight management, especially weight loss among individuals with obesity, is recommended to mitigate the risk of diabetes‐related complications [3]. Diabetes is a major cause of mortality on a global scale, accounting for 1.6 million deaths in 2021. Notably, nearly half of these individuals were aged < 70 years [4]. Uncontrolled or poorly managed diabetes can lead to multi‐organ dysfunction and systemic complications, which significantly increase the risk of diabetes‐related mortality [5]. High blood glucose levels are also responsible for approximately 11% of cardiovascular deaths worldwide, highlighting the profound cardiovascular risks associated with diabetes [4].

Body mass index (BMI) demonstrates a J‐shaped association with all‐cause mortality in the general population, with individuals who are underweight and those with severe obesity exhibiting elevated risks [6]. In a large cohort study investigating the relationship between BMI and mortality among individuals with incident T2D, not only was obesity (BMI ≥ 30 kg/m^2^) associated with increased mortality, but also individuals with a BMI of 18.5–22.4 kg/m^2^ exhibited an elevated risk of mortality compared with that observed in the reference group (BMI 22.5–24.9 kg/m^2^) [7]. Prior population‐based studies have reported U‐shaped associations between BMI and mortality in people with diabetes, and have noted paradoxically lower mortality in overweight or mildly obese individuals compared to their leaner counterparts [8, 9]. However, these studies did not stratify underweight individuals by severity, limiting insight into potential mortality gradients within this vulnerable subgroup.

Most previous studies focused on the effects of obesity on diabetes; however, it is increasingly recognized that not all individuals with diabetes are obese [10]. Racial and ethnic disparities indicate disproportionately high prevalence of diabetes at a lower BMI in non‐white populations [11]. This trend has also been observed among underweight individuals, where T2D may reflect a phenotype marked by reduced skeletal muscle mass and strength—features of sarcopenia—that are disproportionately prevalent in Asian populations and independently associated with insulin resistance and metabolic complications [12]. Nevertheless, the effect of being underweight among individuals with T2D remains an understudied area. Therefore, this study evaluated the effect of being underweight (BMI < 18.5 kg/m^2^) on overall and cause‐specific mortality among individuals with T2D.

## 2 Methods

### 2.1 Korean National Health Insurance Service Database

The Korean National Health Insurance Service (NHIS), a single insurer that manages the National Health Insurance programme, covers approximately 50 million individuals in South Korea. The NHIS database is nationally representative as enrollment is mandatory. The NHIS database has been described in detail elsewhere [13].

NHIS offers a biennial health screening programme for individuals aged ≥ 20 years that collects comprehensive health data, including measurements of body weight and height, blood pressure, waist circumference, smoking status, alcohol consumption, physical activity level and laboratory test results (e.g., complete blood counts, liver and renal function tests, fasting blood glucose levels and lipid profiles) (Supporting Information: Method).

### 2.2 Study Population

Individuals with T2D aged ≥ 40 years who participated in the NHIS biennial health examination between 1 January 2015 and 31 December 2016 and were followed up until 31 December 2022 were included in this study. T2D was defined as documented ICD‐10 codes of E11–E14 with a history of receiving antidiabetic medication before the screening date. Among the 1 886 398 participants initially identified, those aged < 40 years (*n* = 38 711) and individuals with missing critical variables (*n* = 58 691) were excluded. Therefore, 1 788 996 participants were included in the final analysis (Figure S1).

Missing data were handled with complete case analysis. Required variables included BMI, height, weight, waist circumference, blood pressure, smoking, alcohol use, regular exercise, income, fasting glucose, total cholesterol, creatinine, urine protein, alanine aminotransferase and haemoglobin (Table S1). No imputation was performed because overall missingness was low. Because exclusions were based on any missing value, counts by data source (laboratory, questionnaire, anthropometry) are not mutually exclusive.

This study was approved by the Institutional Review Board of the Hallym University Dongtan Sacred Heart Hospital (HDT 2025‐02‐001). Owing to the use of anonymized and de‐identified data, the requirement for obtaining informed consent was waived.

### 2.3 Definition of Underweight

Underweight status, defined as a BMI of < 18.5 kg/m^2^, was categorized into three subgroups: mildly underweight (17.0 ≤ BMI < 18.5 kg/m^2^), moderately underweight (16.0 ≤ BMI < 17.0 kg/m^2^), and severely underweight (BMI < 16.0 kg/m^2^) [14]. Normal weight, overweight and obesity were classified according to the Asia‐Pacific classification [15]: normal weight (18.5 ≤ BMI < 23.0 kg/m^2^), overweight (23.0 ≤ BMI < 25.0 kg/m^2^), class I obesity (25.0 ≤ BMI < 30.0 kg/m^2^), class II obesity (30.0 ≤ BMI < 35.0 kg/m^2^), and class III obesity (BMI ≥ 35.0 kg/m^2^).

### 2.4 Mortality Outcome

All‐cause mortality and mortality due to specific causes were assessed. Cause of death was determined based on the underlying cause recorded in the Korean National Death Registry, classified according to the ICD‐10 code. Diabetes‐related mortality, categorized under ICD codes of E10–E14, included deaths directly linked to diabetes mellitus and its complications. Cardiovascular mortality, categorized under ICD codes of I20–I51, included deaths directly linked to ischemic heart disease, heart failure, arrhythmias and other heart‐related conditions. Cerebrovascular mortality, categorized under ICD codes of I60–I69, included deaths directly linked to stroke and other cerebrovascular diseases.

In cases where multiple conditions were listed, the cause of death was classified according to the primary diagnosis documented on the official death certificate.

### 2.5 Statistical Analysis

Baseline characteristics of the study population were summarized according to the BMI categories. The hazard ratios (HRs) and 95% confidence intervals (CIs) for all‐cause and cause‐specific mortality across BMI categories were estimated using Cox proportional hazards regression models, with the BMI range of ≥ 18.5 kg/m^2^ as the reference group. For the analyses for broader BMI range including obesity categories, the reference point was used as 25.0–29.9 kg/m^2^. This category represented the largest proportion of the study population and has previously been associated with the lowest mortality risk in individuals with type 2 diabetes [16]. Kaplan–Meier method was used for the survival curves. The nonlinear association between BMI and mortality was evaluated using penalized spline regression.

Multivariable models were sequentially adjusted for potential confounders. Model 1 was unadjusted. Model 2 was adjusted for age, sex, socioeconomic factors (income level), lifestyle behaviours (smoking, alcohol consumption and physical activity), and Charlson comorbidity index (CCI). Model 3 was further adjusted for diabetes‐related factors, including the fasting glucose levels, use of ≥ 3 oral antidiabetic medications or insulin, and duration of diabetes. Model 4 additionally adjusted for haemoglobin and alanine aminotransferase.

Stratified analyses were conducted to evaluate the interactions between BMI and key demographic and clinical subgroups, including age, sex and comorbidities. The interaction terms were tested using likelihood ratio tests to assess effect modification.

To account for the potential confounding effect of cancer‐related cachexia and non‐health‐related deaths (e.g., trauma), competing risk analyses were performed as part of the sensitivity analysis. Cancer‐related deaths were identified using ICD‐10 codes C00–C97, and accidental or trauma‐related deaths using codes S00–T98; these were treated as competing events when estimating cause‐specific mortality.

Additional sensitivity analyses were conducted using lag periods of 1 and 2 years, excluding individuals who died within the first 1 or 2 years of follow‐up, respectively, to mitigate the risk of reverse causality.

All statistical analyses were performed using SAS version 9.4 (SAS Institute Inc., Cary, NC, USA). Statistical significance is set at a two‐sided *p* value of < 0.05.

## 3 Results

### 3.1 Baseline Characteristics of the Study Participants

In total, 1 788 996 participants with T2D were followed up for a median duration of 7.0 years (IQR 6.3–7.4 years) (Table 1). The mean BMI was 25.2 kg/m^2^, with the majority (41.0%) in the BMI range of 25.0–29.9 kg/m^2^ (Table S2). Notably, the BMI of 1920, 3866 and 18 554 patients was < 16.0 kg/m^2^, 16.0–16.9 kg/m^2^, and 17.0–18.4 kg/m^2^, respectively.

**TABLE 1:** Baseline characteristics of the study participants stratified according to underweight status.

*N* (%)	Total	BMI groups				*p* value
		Underweight (+)			Underweight (−)	
		Severe	Moderate	Mild		
		< 16.0 kg/m^2^	16.0–16.9 kg/m^2^	17.0–18.4 kg/m^2^	≥ 18.5 kg/m^2^	
	1 788 996 (100%)	1920 (0.1%)	3866 (0.2%)	18 554 (1.0%)	1 764 656 (98.6%)	
***Demographics and laboratory parameters***						
BMI (kg/m^2^)	25.2 ± 3.4	15.1 ± 0.8	16.5 ± 0.3	17.9 ± 0.4	25.3 ± 3.3	< 0.0001
Height (cm)	161.7 ± 9.2	159.2 ± 9.4	159.5 ± 9.2	160.1 ± 9.1	161.7 ± 9.2	< 0.0001
Weight (kg)	66.0 ± 11.8	38.4 ± 5.0	42.2 ± 4.9	45.9 ± 5.3	66.3 ± 11.6	< 0.0001
Waist circumference	86.1 ± 8.8	66.3 ± 7.5	68.0 ± 6.5	70.4 ± 5.9	86.3 ± 8.6	< 0.0001
Central obesity	744 495 (41.6)	29 (1.51)	38 (0.98)	195 (1.05)	744 233 (42.2)	< 0.0001
Age (years)	62.5 ± 10.4	68.5 ± 12.8	67.0 ± 12.0	65.6 ± 11.6	62.4 ± 10.4	< 0.0001
≥ 65	745 117 (41.7)	1156 (60.2)	2184 (56.5)	9659 (52.1)	732 118 (41.5)	
Male (*n*, %)	1 010 970 (56.5)	971 (50.6)	2006 (51.9)	9639 (52.0)	998 354 (56.6)	< 0.0001
Systolic blood pressure (mmHg)	128 ± 15	119 ± 17	120 ± 17	122 ± 17	128 ± 15	< 0.0001
Diastolic blood pressure (mmHg)	77 ± 10	73 ± 11	73 ± 10	73 ± 10	77 ± 10	< 0.0001
Fasting glucose (mg/dL)	141 ± 47	149 ± 83	148 ± 76	147 ± 69	141 ± 47	< 0.0001
Total cholesterol (mg/dL)	176 ± 41	171 ± 43	171 ± 41	172 ± 40	176 ± 41	< 0.0001
Triglyceride^a^ (mg/dL)	130 (130, 130)	96 (94, 98)	93 (92, 95)	94 (94, 95)	131 (131, 131)	< 0.0001
HDL‐cholesterol (mg/dL)	50 ± 14	58 ± 19	58 ± 22	57 ± 17	50 ± 14	< 0.0001
LDL‐cholesterol (mg/dL)	96 ± 36	92 ± 36	92 ± 35	94 ± 35	96 ± 36	< 0.0001
eGFR (mL/min/1.73 m^2^)	81.5 ± 20.1	80.4 ± 22.7	80.8 ± 22.6	81.6 ± 21.5	81.5 ± 20.0	0.0225
***Social history***						
Smoking (*n*, %)						< 0.0001
Never smoker	1 045 447 (58.4)	1191 (62.0)	2336 (60.4)	11 165 (60.2)	1 030 755 (58.4)	
Ex‐smoker	403 770 (22.6)	243 (12.7)	563 (14.6)	2884 (15.5)	400 080 (22.7)	
Current smoker	339 779 (19.0)	486 (25.3)	967 (25.0)	4505 (24.3)	333 821 (18.9)	
Alcohol (*n*, %)						< 0.0001
None	1 150 169 (64.3)	1524 (79.4)	2997 (77.5)	13 718 (73.9)	1 131 930 (64.1)	
Mild	507 857 (28.4)	296 (15.4)	681 (17.6)	3738 (20.2)	503 142 (28.5)	
Heavy	130 970 (7.32)	100 (5.21)	188 (4.86)	1098 (5.92)	129 584 (7.34)	
Regular exercise (*n*, %)	398 108 (22.3)	197 (10.3)	604 (15.6)	3475 (18.7)	393 832 (22.3)	< 0.0001
Low income (*n*, %)	393 655 (22.0)	518 (27.0)	1033 (26.7)	4739 (25.5)	387 365 (23.0)	< 0.0001
***Medical history***						
Hypertension (*n*, %)	1 165 363 (65.1)	901 (46.9)	1821 (47.1)	8608 (46.4)	1 154 033 (65.4)	< 0.0001
Dyslipidaemia (*n*, %)	1 179 321 (65.9)	758 (39.5)	1596 (41.3)	8586 (46.3)	1 168 381 (66.2)	< 0.0001
Chronic kidney disease (*n*, %)	241 919 (13.5)	353 (18.4)	675 (17.5)	2908 (15.7)	237 983 (13.5)	< 0.0001
Duration of diabetes (years)						< 0.0001
New onset	130 737 (7.31)	164 (8.54)	255 (6.60)	1156 (6.23)	129 162 (7.32)	
< 5 years	547 892 (30.6)	493 (25.7)	928 (24.0)	4384 (23.6)	542 087 (30.7)	
< 10 years	491 263 (27.5)	484 (25.2)	963 (24.9)	4632 (25.0)	485 184 (27.5)	
≥ 10 years	619 104 (34.6)	779 (40.6)	1720 (44.5)	8382 (45.2)	608 223 (34.5)	
Use of ≥ 3 oral antidiabetic drugs	557 159 (31.1)	512 (26.7)	1166 (30.2)	5695 (30.7)	549 786 (31.2)	< 0.0001
Use of insulin (*n*, %)	198 188 (11.1)	418 (21.8)	830 (21.5)	3557 (19.2)	193 383 (11.0)	< 0.0001
**Charlson comorbidity index**	3.46 ± 2.15	4.05 ± 2.45	4.01 ± 2.44	3.83 ± 2.38	3.45 ± 2.15	< 0.0001
Myocardial infarction (*n*, %)	28 164 (1.57)	43 (2.24)	72 (1.86)	321 (1.73)	27 728 (1.57)	0.0014
Congestive heart failure (*n*, %)	74 518 (4.17)	120 (6.25)	244 (6.31)	919 (4.95)	73 235 (4.15)	< 0.0001
Peripheral vascular disease (*n*, %)	375 282 (21.0)	407 (21.2)	861 (22.3)	4121 (22.2)	369 893 (21.0)	0.0002
Cerebrovascular disease (*n*, %)	239 548 (13.4)	403 (21.0)	750 (19.4)	3021 (16.3)	235 374 (13.3)	< 0.0001
Dementia (*n*, %)	51 486 (2.88)	344 (17.9)	431 (11.2)	1407 (7.58)	49 304 (2.79)	< 0.0001
Chronic pulmonary disease (*n*, %)	532 940 (29.8)	699 (36.4)	1364 (35.3)	5841 (31.5)	525 036 (29.8)	< 0.0001
Rheumatologic disease (*n*, %)	81 981 (4.58)	117 (6.09)	208 (5.38)	1015 (5.47)	80 641 (4.57)	< 0.0001
Peptic ulcer disease (*n*, %)	508 638 (28.4)	646 (33.7)	1240 (32.1)	5739 (30.9)	501 013 (28.4)	< 0.0001
Mild liver disease (*n*, %)	704 493 (39.4)	760 (39.6)	1562 (40.4)	7267 (39.2)	694 904 (39.4)	0.6396
Moderate/severe liver disease	9744 (0.54)	19 (0.99)	39 (1.01)	170 (0.92)	9516 (0.54)	< 0.0001
Hemiplegia or paraplegia (*n*, %)	10 637 (0.59)	47 (2.45)	52 (1.35)	232 (1.25)	10 306 (0.58)	< 0.0001
Any malignancy (*n*, %)	283 991 (15.9)	353 (18.4)	764 (19.8)	3515 (18.9)	279 359 (15.8)	< 0.0001
Metastatic solid tumour (*n*, %)	9145 (0.51)	22 (1.15)	38 (0.98)	181 (0.98)	8904 (0.50)	< 0.0001
AIDS/HIV (*n*, %)	39 (0)	0 (0)	0 (0)	0 (0)	39 (0)	0.5065

*Note:* Data are presented as mean ± standard deviation (SD) and *n* (%) for continuous and categorical variables, respectively. *p* value indicates a test for trend.

Abbreviations: BMI, body mass index; CVD, cardiovascular disease; eGFR, estimated glomerular filtration rate; HDL, high‐density lipoprotein; LDL, low‐density lipoprotein.

^a^ Triglycerides are presented as geometric mean (95% confidence interval) after log transformation due to right‐skewed distribution.

Participants who were underweight were generally older, were more likely to be current smokers, and were more likely to belong to the low‐income group. The proportion of these individuals engaging in regular exercise was lower. Alcohol consumption was higher among participants who were not underweight.

At baseline, 7.3% of participants were newly diagnosed with T2D. The duration of diabetes was < 5 years, 5–10 years, and ≥ 10 years in 30.6%, 27.5% and 34.6% of participants, respectively. A total of 557 159 participants (31.1%) were receiving ≥ 3 oral antidiabetic agents, whereas 198 188 (11.1%) were using insulin. A higher proportion of participants who were underweight were using insulin, whereas participants with obesity were more likely to be receiving ≥ 3 oral antidiabetic agents. The mean fasting glucose levels across the cohort were 141 ± 47 mg/dL. The mean glucose levels of participants with lower BMI were significantly higher than those of the participants with a BMI of ≥ 18.5 kg/m^2^ (*p* < 0.0001).

The prevalence of comorbidities, such as hypertension (65.1%), dyslipidaemia (65.9%) and chronic kidney disease (13.5%), was high among the study participants. The prevalence of hypertension and dyslipidaemia increased as the BMI increased, whereas CKD was more prevalent among the participants with lower BMI.

### 3.2 Increased Overall Mortality Among the Participants With Type 2 Diabetes Who Were Underweight

**FIGURE 1:**
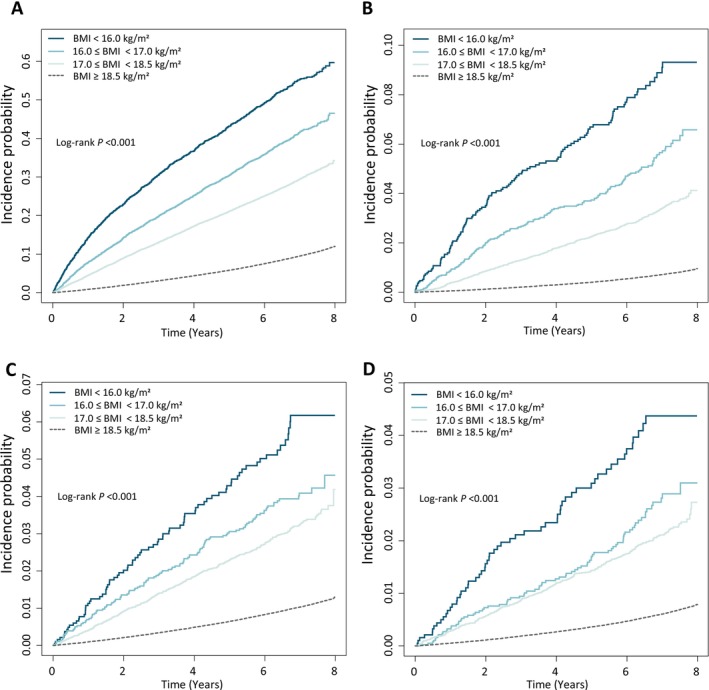
Cumulative incidence of mortality across body mass index (BMI) categories among individuals with type 2 diabetes. (A) All‐cause mortality, (B) Diabetes‐related mortality, (C) Cardiovascular disease‐related mortality and (D) Cerebrovascular disease‐related mortality. BMI categories were divided into severely underweight (< 16.0 kg/m^2^), moderately underweight (16.0–16.9 kg/m^2^), mildly underweight (17.0–18.4 kg/m^2^) and BMI ≥ 18.5 kg/m^2^.

Over the median follow‐up period, 176 056 (9.8%) deaths from all causes were observed. The risk of all‐cause mortality among individuals who were underweight was significantly higher than that among those with a BMI of ≥ 18.5 kg/m^2^ (Figure S2). Mortality risk was elevated in the mildly underweight subgroup (BMI 17.0–18.4 kg/m^2^; HR, 3.465; 95% CI, 3.378–3.554); further increase in mortality risk was observed in the moderately underweight subgroup (BMI 16.0–16.9 kg/m^2^; HR, 5.157; 95% CI, 4.922–5.405). The highest risk was observed in the severely underweight subgroup (BMI < 16.0 kg/m^2^; HR, 7.616; 95% CI, 7.182–8.978) (Figure 1 and Table 2).

**TABLE 2:** Stepwise multivariable adjustments for BMI in underweight participants and mortality associations.

Outcome	BMI groups (kg/m^2^)	N	Event	Duration	Model 1	Model 2	Model 3	Model 4
All‐cause mortality	< 16.0	1920	1049	9067	8.630 (8.122–9.170)	5.041 (4.744–5.357)	4.679 (4.402–4.973)	3.876 (3.646–4.119)
	16.0–16.9	3866	1595	21 066	5.524 (5.258–5.803)	3.455 (3.289–3.630)	3.197 (3.043–3.360)	2.719 (2.587–2.857)
	17.0–18.4	18 554	5460	110 313	3.555 (3.461–3.653)	2.501 (2.434–2.569)	2.319 (2.256–2.383)	2.037 (1.982–2.094)
	≥ 18.5	1 764 656	167 952	11 822 519	1 (ref.)	1 (ref.)	1 (ref.)	1 (ref.)
	*p*				< 0.0001	< 0.0001	< 0.0001	< 0.0001
Diabetes mortality	< 16.0	1920	125	9067	14.42 (12.09–17.20)	7.901 (6.622–9.428)	6.913 (5.790–8.254)	5.136 (4.300–6.134)
	16.0–16.9	3866	176	21 066	8.510 (7.333–9.875)	4.993 (4.301–5.796)	4.232 (3.642–4.917)	3.306 (2.845–3.843)
	17.0–18.4	18 554	537	110 313	4.867 (4.464–5.307)	3.280 (3.008–3.578)	2.766 (2.533–3.021)	2.265 (2.073–2.474)
	≥ 18.5	1 764 656	12 115	11 822 519	1 (ref.)	1 (ref.)	1 (ref.)	1 (ref.)
	*p*				< 0.0001	< 0.0001	< 0.0001	< 0.0001
Cardiovascular disease mortality	< 16.0	1920	80	9067	6.253 (5.020–7.788)	3.398 (2.727–4.234)	3.315 (2.661–4.129)	2.825 (2.267–3.519)
	16.0–16.9	3866	127	21 066	4.194 (3.522–4.994)	2.483 (2.085–2.957)	2.394 (2.009–2.853)	2.087 (1.751–2.487)
	17.0–18.4	18 554	521	110 313	3.245 (2.974–3.540)	2.200 (2.016–2.401)	2.105 (1.927–2.300)	1.881 (1.721–2.055)
	≥ 18.5	1 764 656	17 495	11 822 519	1 (ref.)	1 (ref.)	1 (ref.)	1 (ref.)
	*p*				< 0.0001	< 0.0001	< 0.0001	< 0.0001
Cerebrovascular disease mortality	< 16.0	1920	58	9067	7.838 (6.057–10.142)	4.211 (3.253–5.453)	3.907 (3.016–5.061)	3.501 (2.702–4.537)
	16.0–16.9	3866	81	21 066	4.590 (3.689–5.712)	2.734 (2.196–3.403)	2.521 (2.024–3.140)	2.300 (1.846–2.866)
	17.0–18.4	18 554	339	110 313	3.614 (3.244–4.027)	2.461 (2.208–2.743)	2.271 (2.035–2.535)	2.100 (1.881–2.344)
	≥ 18.5	1 764 656	10 281	11 822 519	1 (ref.)	1 (ref.)	1 (ref.)	1 (ref.)
	*p*				< 0.0001	< 0.0001	< 0.0001	< 0.0001

*Note:* Data are presented as hazard ratios (95% confidence intervals). Model adjustments: Model 1, unadjusted; Model 2, age, sex, income level, smoking status, alcohol consumption, physical activity and the Charlson comorbidity index; Model 3, Model 2 + fasting glucose, use of ≥ 3 oral antidiabetic medications or insulin and duration of diabetes; Model 4, Model 3 + haemoglobin and alanine aminotransferase.

Abbreviation: BMI, body mass index.

This trend persisted after age and sex (Model 2) were adjusted for and remained consistent after social history, comorbidities (Model 3), and diabetes‐specific factors (e.g., use of ≥ 3 oral antidiabetic agents, insulin and duration of diabetes) were adjusted for (Model 4). (Table 2).

### 3.3 Subgroup Analysis for Overall Mortality Among the Participants With Type 2 Diabetes Who Were Underweight

**FIGURE 2:**
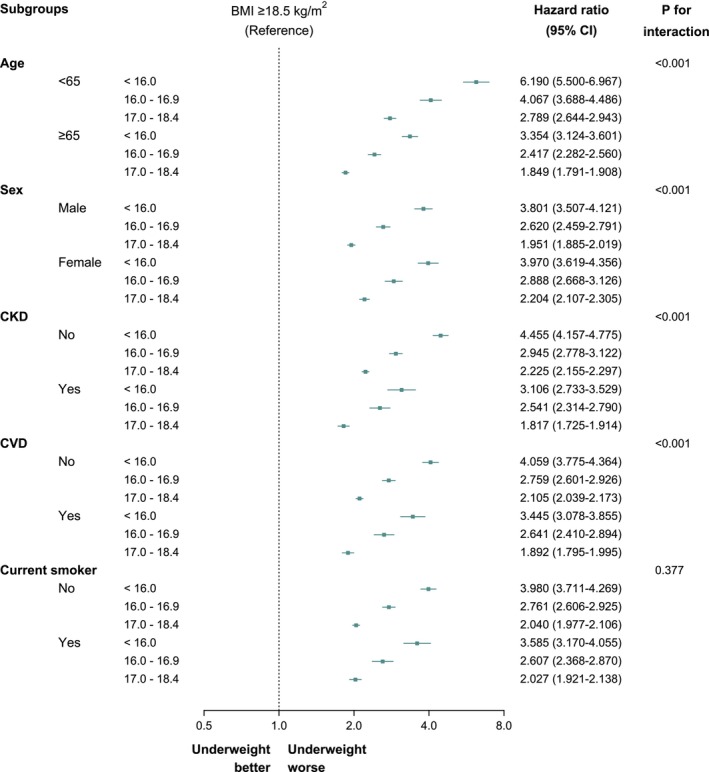
Subgroup analysis of all‐cause mortality across body mass index (BMI) categories among individuals with type 2 diabetes. Hazard ratios (HRs) and 95% confidence intervals (CIs) for all‐cause mortality are shown for the following BMI groups: < 16.0 kg/m^2^, 16.0–16.9 kg/m^2^, 17.0–18.4 kg/m^2^ and ≥ 18.5 kg/m^2^ (reference group) across subgroups of age, sex, chronic kidney disease (CKD), history of cardiovascular disease (CVD), central obesity and smoking status. All estimates are based on the fully adjusted Model 4, which includes age, sex, income level, smoking status, alcohol consumption, physical activity, Charlson comorbidity index (CCI), fasting glucose levels, use of ≥ 3 oral antidiabetic medications or insulin, duration of diabetes, haemoglobin and alanine aminotransferase.

A subgroup analysis using the fully adjusted model was performed to examine the effect of BMI on overall mortality among the participants with T2D with various demographic and clinical characteristics (Figure 2 and Table S3). A consistent and significant increase in all‐cause mortality risk was observed among participants with T2D who were underweight compared with that observed among those with a BMI of ≥ 18.5 kg/m^2^. Variations were observed across subgroups.

Age‐stratified analyses revealed consistently elevated mortality risks associated with underweight status in both younger (< 65 years) and older (≥ 65 years) adults with type 2 diabetes. Although the absolute mortality rate was higher in the older age group, the relative risk associated with severe underweight (BMI < 16.0 kg/m^2^) was greater in those aged < 65 years (aHR, 6.190; 95% CI, 5.500–6.967), compared to those aged ≥ 65 years (aHR, 3.354; 95% CI, 3.124–3.601) (*p* for interaction < 0.001).

The presence of CKD or cardiovascular disease was associated with elevated mortality risk across all BMI groups. The absolute mortality rate among participants with CKD or cardiovascular disease was significantly higher; nevertheless, being severely underweight remained a strong independent risk factor even in their absence. A substantial mortality risk (HR, 3.106; 95% CI, 2.733–3.529) was observed among the participants with CKD who were severely underweight; however, an even higher mortality risk (HR, 4.455; 95% CI, 4.157–4.775) was observed among those without CKD (*p* for interaction < 0.001). Similarly, participants with a history of cardiovascular disease were associated with an elevated risk of mortality among participants who were severely underweight (HR, 3.445; 95% CI, 3.078–3.855); an even greater increase in risk (HR, 4.059; 95% CI, 3.775–4.364) was observed among the participants without cardiovascular disease (*p* for interaction < 0.001).

Although the interaction term for smoking status was not statistically significant (*p* for interaction = 0.377), participants with T2D who were underweight and non‐smokers or ex‐smokers had higher mortality risks than those who were current smokers.

### 3.4 Increased All‐Cause and Cause‐Specific Mortality Across BMI Categories Among the Participants With Type 2 Diabetes

**FIGURE 3:**
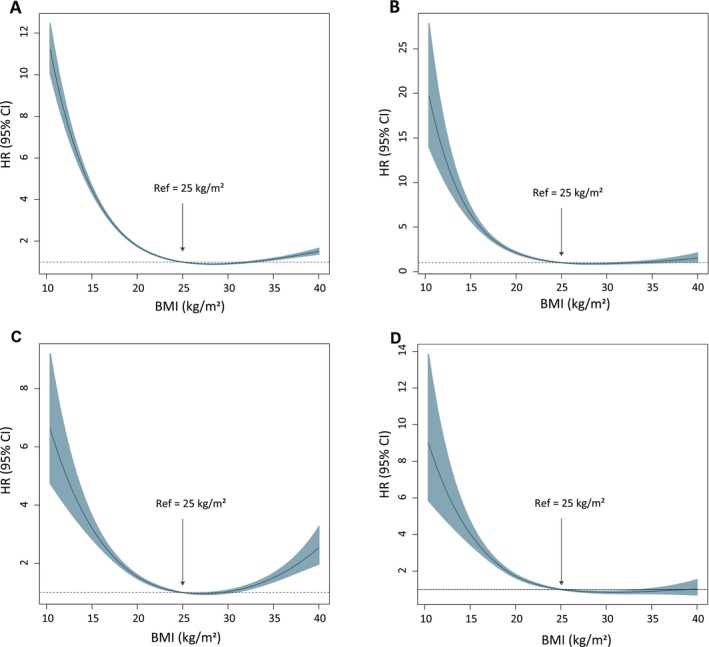
Penalized spline regression curves for the association between body mass index (BMI) and mortality among individuals with type 2 diabetes. Hazard ratios (HRs) and 95% confidence intervals (CIs) are depicted for (A) all‐cause mortality, (B) diabetes‐related mortality, (C) cardiovascular disease‐related mortality and (D) cerebrovascular disease‐related mortality. The solid lines represent HRs and the shaded areas represent the 95% CIs. The arrows indicate the reference point set at BMI 25.0 kg/m^2^. The model is adjusted for age, sex, income level, smoking status, alcohol consumption, physical activity, Charlson comorbidity index (CCI), fasting glucose levels, use of ≥ 3 oral antidiabetic medications or insulin, duration of diabetes, haemoglobin and alanine aminotransferase.

A strong association was observed between underweight status and overall mortality. Therefore, all‐cause and cause‐specific mortality across all BMI groups was examined further, with a special focus on diabetes‐related (12 953 deaths), cardiovascular disease‐related (18 223 deaths) and cerebrovascular disease‐related (10 759 deaths) deaths among individuals with T2D (Figure 3 and Table 3).

**TABLE 3:** Comprehensive BMI categories and mortality associations with stepwise multivariable adjustments.

Outcome	BMI group	N	Event	Duration	Model 1	Model 2	Model 3	Model 4
All‐cause mortality	< 16.0	1920	1049	9067	10.885 (10.240–11.571)	6.043 (5.684–6.424)	6.420 (6.034–6.830)	5.168 (4.857–5.499)
	16.0–16.9	3866	1595	21 066	6.966 (6.628–7.322)	4.138 (3.936–4.349)	4.393 (4.175–4.622)	3.623 (3.443–3.812)
	17.0–18.4	18 554	5460	110 313	4.484 (4.361–4.610)	2.986 (2.904–3.071)	3.178 (3.086–3.273)	2.706 (2.627–2.787)
	18.5–22.9	437 385	60 503	2 869 030	1.878 (1.856–1.899)	1.526 (1.509–1.544)	1.648 (1.625–1.672)	1.544 (1.522–1.566)
	23.0–24.9	446 154	42 056	2 994 821	1.245 (1.230–1.261)	1.104 (1.090–1.118)	1.168 (1.152–1.184)	1.148 (1.132–1.164)
	25.0–29.9	733 354	55 947	4 958 909	1 (ref.)	1 (ref.)	1 (ref.)	1 (ref.)
	30.0–34.9	129 823	8296	878 899	0.838 (0.819–0.857)	1.109 (1.084–1.135)	1.070 (1.045–1.095)	1.077 (1.052–1.103)
	≥ 35.0	17 940	1150	120 860	0.847 (0.799–0.898)	1.572 (1.483–1.667)	1.511 (1.425–1.602)	1.504 (1.418–1.595)
	*p*				< 0.0001	< 0.0001	< 0.0001	< 0.0001
Diabetes‐related mortality	< 16.0	1920	125	9067	20.526 (17.177–24.528)	10.696 (8.944–12.792)	11.161 (9.299–13.395)	7.784 (6.482–9.348)
	16.0–16.9	3866	176	21 066	12.076 (10.380–14.049)	6.754 (5.803–7.861)	6.851 (5.860–8.009)	5.016 (4.288–5.868)
	17.0–18.4	18 554	537	110 313	6.906 (6.307–7.562)	4.419 (4.034–4.841)	4.461 (4.044–4.921)	3.416 (3.095–3.770)
	18.5–22.9	437 385	5028	2 869 030	2.435 (2.333–2.542)	1.966 (1.883–2.053)	2.056 (1.948–2.169)	1.820 (1.724–1.921)
	23.0–24.9	446 154	2882	2 994 821	1.331 (1.267–1.398)	1.189 (1.132–1.249)	1.249 (1.184–1.316)	1.203 (1.141–1.268)
	25.0–29.9	733 354	3588	4 958 909	1 (ref.)	1 (ref.)	1 (ref.)	1 (ref.)
	30.0–34.9	129 823	533	878 899	0.840 (0.767–0.920)	1.066 (0.973–1.168)	1.046 (0.954–1.147)	1.070 (0.976–1.173)
	≥ 35.0	17 940	84	120 860	0.966 (0.778–1.199)	1.643 (1.323–2.041)	1.600 (1.288–1.988)	1.610 (1.296–2.000)
	*p*				< 0.0001	< 0.0001	< 0.0001	< 0.0001
Cardiovascular disease‐related mortality	< 16.0	1920	80	9067	7.595 (6.092–9.469)	3.896 (3.125–4.856)	4.150 (3.322–5.183)	3.413 (2.732–4.264)
	16.0–16.9	3866	127	21 066	5.094 (4.273–6.073)	2.828 (2.372–3.373)	2.999 (2.509–3.586)	2.520 (2.108–3.014)
	17.0–18.4	18 554	521	110 313	3.941 (3.604–4.310)	2.502 (2.287–2.737)	2.633 (2.395–2.894)	2.267 (2.061–2.493)
	18.5–22.9	437 385	5934	2 869 030	1.702 (1.642–1.764)	1.356 (1.308–1.406)	1.440 (1.378–1.506)	1.346 (1.287–1.407)
	23.0–24.9	446 154	4364	2 994 821	1.195 (1.150–1.243)	1.053 (1.013–1.095)	1.104 (1.058–1.151)	1.080 (1.035–1.127)
	25.0–29.9	733 354	6049	4 958 909	1 (ref.)	1 (ref.)	1 (ref.)	1 (ref.)
	30.0–34.9	129 823	976	878 899	0.911 (0.852–0.975)	1.203 (1.124–1.287)	1.172 (1.095–1.255)	1.192 (1.113–1.276)
	≥ 35.0	17 940	172	120 860	1.170 (1.006–1.362)	2.160 (1.856–2.515)	2.096 (1.800–2.442)	2.119 (1.819–2.468)
	*p*				< 0.0001	< 0.0001	< 0.0001	< 0.0001
Cerebrovascular disease‐related mortality	< 16.0	1920	58	9067	9.867 (7.612–12.791)	5.043 (3.889–6.539)	5.390 (4.145–7.008)	4.731 (3.637–6.154)
	16.0–16.9	3866	81	21 066	5.794 (4.648–7.222)	3.270 (2.622–4.078)	3.484 (2.785–4.359)	3.108 (2.483–3.890)
	17.0–18.4	18 554	339	110 313	4.556 (4.075–5.094)	2.935 (2.624–3.284)	3.132 (2.782–3.525)	2.828 (2.511–3.186)
	18.5–22.9	437 385	3735	2 869 030	1.896 (1.810–1.986)	1.526 (1.457–1.599)	1.640 (1.549–1.737)	1.562 (1.474–1.655)
	23.0–24.9	446 154	2635	2 994 821	1.276 (1.213–1.343)	1.136 (1.079–1.195)	1.196 (1.132–1.264)	1.175 (1.112–1.242)
	25.0–29.9	733 354	3421	4 958 909	1 (ref.)	1 (ref.)	1 (ref.)	1 (ref.)
	30.0–34.9	129 823	442	878 899	0.730 (0.661–0.806)	0.941 (0.852–1.039)	0.912 (0.825–1.008)	0.927 (0.839–1.025)
	≥ 35.0	17 940	48	120 860	0.578 (0.435–0.769)	1.023 (0.769–1.360)	4.150 (3.322–5.183)	3.413 (2.732–4.264)
	*p*				< 0.0001	< 0.0001	< 0.0001	< 0.0001

*Note:* BMI categories are defined as follows: severe underweight, < 16.0 kg/m^2^; moderate underweight, 16.0–16.9 kg/m^2^; mild underweight, 17.0–18.4 kg/m^2^; normal weight, 18.5–22.9 kg/m^2^; overweight, 23.0–24.9 kg/m^2^; Class I Obesity (reference group) 25.0–29.9 kg/m^2^; Class II Obesity, 30.0–34.9 kg/m^2^; and Class III Obesity, ≥ 35 kg/m^2^. Hazard ratios (HRs) are presented with 95% confidence intervals (CIs). Model adjustments: Model 1, unadjusted; Model 2, age, sex, income level, smoking status, alcohol consumption, physical activity and the Charlson comorbidity index; Model 3, Model 2 + fasting glucose, use of ≥ 3 oral antidiabetic medications or insulin and duration of diabetes; Model 4, Model 3 + haemoglobin and alanine aminotransferase.

Abbreviations: BMI, body mass index; IR, incidence rates.

Compared with that observed in the reference group (BMI 25.0–29.9 kg/m^2^, class I obesity), the mortality risk among individuals who were underweight was significantly higher. The risk of all‐cause mortality progressively increased with the severity of underweight status in the fully adjusted model: mildly underweight (HR, 2.706; 95% CI, 2.627–2.787), moderately underweight (HR, 3.623; 95% CI, 3.443–3.812) and severely underweight (HR, 5.168; 95% CI, 4.857–5.499). Notably, the mortality risk among individuals with a normal BMI (18.5–22.9 kg/m^2^; HR, 1.544; 95% CI, 1.522–1.566) and those classified as overweight (BMI 23.0–24.9 kg/m^2^; HR, 1.148; 95% CI, 1.132–1.164) was also elevated compared with that observed in the reference group.

Class II and III obesity (BMI ≥ 30 kg/m^2^) appeared to exert a protective effect in the crude model; however, this relationship reversed after adjusting for age and sex. Class III obesity (BMI ≥ 35 kg/m^2^) was associated with a modest but significant increase in mortality risk (HR, 1.504; 95% CI, 1.418–1.595) compared to the reference group in the fully adjusted model, though this risk was substantially lower than that observed in underweight groups.

Diabetes‐related mortality exhibited a comparable trend, with individuals who were severely underweight exhibiting the most pronounced effect (HR, 7.784; 95% CI, 6.482–9.348) in the fully adjusted model. Normal BMI was also associated with an increase in mortality risk (HR, 1.820; 95% CI, 1.724–1.92); however, class II obesity (BMI 30.0–34.9 kg/m^2^) exhibited no significant association (HR, 1.070; 95% CI, 0.976–1.173). Class III obesity (BMI ≥ 35.0 kg/m^2^) was associated with an increase in diabetes‐related mortality risk (HR, 1.610; 95% CI, 1.296–2.000).

Cardiovascular disease‐related mortality exhibited a similar pattern in the fully adjusted model, with individuals who were underweight exhibiting elevated risks. Cerebrovascular disease‐related mortality was also elevated among individuals who were underweight; however, no significant association was observed for class III obesity (BMI ≥ 35.0 kg/m^2^) compared with the reference group (HR, 0.927; 95% CI, 0.754–1.334).

### 3.5 Sensitivity Analysis

To address the potential confounding effects of cancer‐related cachexia and accidental deaths, we performed competing risk analyses treating cancer‐related and trauma‐related deaths as competing events (Table S4). In these models, all‐cause mortality remained highest in the group with BMI < 16.0 kg/m^2^. After full adjustment, the risk for all‐cause mortality was increased in BMI < 16.0 kg/m^2^ (HR, 3.843; 95% CI, 3.555–4.154), in BMI 16.0–16.9 kg/m^2^ (HR, 2.705; 95% CI, 2.551–2.868) and BMI 17.0–18.4 kg/m^2^ (HR, 2.032; 95% CI, 1.970–2.096), compared to the reference group (BMI ≥ 18.5 kg/m^2^).

To mitigate the possibility of reverse causality due to pre‐existing severe illness, we conducted additional analyses excluding deaths that occurred within the first year (1‐year lag) and the first 2 years (2‐year lag) of follow‐up (Tables S5–S6). The results were consistent with the main findings: underweight status remained significantly associated with elevated risk of mortality across all BMI categories.

## 4 Discussion

The present study demonstrated that significantly increased mortality was observed among individuals with T2D who were underweight, with risks substantially exceeding those seen in morbid obesity. This trend was observed for all‐cause and disease‐specific mortalities, including diabetes, cardiovascular and cerebrovascular‐related mortalities. The severity of underweight status was associated with an increasing mortality risk across all causes among the participants who were underweight. The nadir for mortality was observed among individuals with class I obesity (BMI 25.0–29.9 kg/m^2^). However, participants with a normal BMI (18.5–22.9 kg/m^2^) and participants with a BMI of 23.0–24.9 kg/m^2^ exhibited elevated mortality risks. Class II and III obesity (BMI ≥ 30.0 kg/m^2^) appeared to exert a protective effect in the crude models; however, this association was not observed after adjusting for confounders such as age and sex.

The association between underweight status and mortality risk was particularly pronounced among younger individuals and those without CKD or a history of cardiovascular disease. The baseline mortality risk was lower in these subgroups; however, the mortality risk was significantly amplified among participants who were underweight even in the absence of traditional risk factors. While prior studies have shown a stronger link between underweight and mortality among current smokers [17, 18], our analysis found no significant interaction between smoking status and mortality risk. Notably, the mortality rates were higher among underweight non‐smokers, possibly owing to their underweight status not being attributable to smoking‐related weight loss and the influence of survivor bias [19, 20].

Analysis of cause‐specific mortality revealed that diabetes‐related mortality increased sharply with the severity of underweight status among individuals with T2D. Over a tenfold increase in the risk of diabetes‐related mortality was observed among participants who were severely underweight. This pattern was not observed in previous studies that examined BMI and diabetes‐related mortality in the general population [21, 22]. The prevalence of T2D increases with BMI and underweight individuals in the general population have a relatively low prevalence of T2D [23], which may have obscured this association in past analyses. However, the present study, which focused exclusively on individuals with T2D, revealed a dramatic increase in diabetes‐related mortality as the BMI decreased.

Obesity is an established risk factor for diabetes and cardiovascular mortality, and their coexistence significantly amplifies the risk of cardiovascular death [24]. A meta‐analysis of 21 cohort studies revealed that each 5‐unit increase in BMI was associated with a 9% higher risk of cardiovascular disease and mortality among individuals with a BMI of > 28.4 kg/m^2^ [25]. However, each 5‐unit decrease corresponded to a 13% higher risk among those with a BMI of ≤ 28.4 kg/m^2^ [25], indicating that both obesity and body weight below a certain threshold contribute to cardiovascular mortality risk. Similarly, studies in Ukraine and Korea reported higher cardiovascular mortality risk in underweight individuals with diabetes, particularly males with BMI < 25 kg/m^2^ and females with BMI < 23 kg/m^2^ [26, 27]. In a Chinese cohort of 11 449 adults with T2D, a U‐shaped association was observed between BMI and all‐cause mortality, with elevated risks at both extremes [9]. In another study using two large Taiwanese cohorts comprising 61 574 adults with T2D, it was reported that an obesity paradox exists, where lower BMI was associated with increased hospitalization and mortality related to both expanded and non‐expanded cardiovascular diseases [8].

Unlike cardiovascular and diabetes‐related mortality, which demonstrates increased risks at both extremes of BMI, stroke mortality was primarily elevated in underweight and normal‐weight individuals. Notably, class III obesity did not significantly alter stroke mortality risk compared to the reference group. Previous studies highlighted an obesity paradox, where obese patients demonstrated improved survival after stroke [28, 29]. For example, the Korean Stroke Registry reported paradoxical longevity in obese patients post‐stroke and a prospective study of 29 554 individuals with T2D found an inverse association between BMI and stroke risk, even among those with extreme obesity (BMI ≥ 40 kg/m^2^) [30]. In contrast, the present study specifically examined underweight status and stroke‐related mortality in individuals with T2D. We found that severe underweight significantly increases stroke‐related mortality, while obesity may offer protection.

The increased mortality risk observed among individuals with T2D who are underweight may be attributed to a combination of biological, metabolic and systemic factors [31]. Greater lean muscle mass is more metabolically protective than adipose tissue and contributes to a higher BMI, while excess adiposity in older adults may confer a survival advantage [32]. In our study, a flipped J‐shaped association was observed, with underweight participants who were generally older, had a lower socioeconomic status, and longer diabetes duration exhibiting the highest mortality risk. A small subset of severely underweight individuals with central obesity demonstrated features consistent with sarcopenic obesity—characterized by reduced muscle mass, increased visceral fat and a high burden of comorbidities. This phenotype may reflect sarcopenia, malnutrition and advanced disease states, which impair glucose metabolism, increase insulin resistance and worsen glycemic control [33]. Genetic susceptibility among individuals with T2D who are underweight may also contribute to the increased mortality risk, differentiating them from their counterparts who are obese [34].

Lean diabetes and a higher susceptibility to obesity‐related complications at lower BMI levels is observed among Asians [35]. However, the average BMI of the participants with T2D was 25.3 ± 3.5 kg/m^2^ in this study, classified as class I obesity under the Asia‐Pacific WHO criteria [15]. This is consistent with the findings from the Action to Control Cardiovascular Risk in Diabetes trial [36] and the Bezafibrate Infarction Prevention study, which demonstrated a BMI nadir for mortality around 25–30 kg/m^2^ for individuals with diabetes [37]. These results suggest that modern lifestyle habits and the rising prevalence of diabetes and obesity among East Asians may have shifted their metabolic profile closer to that of Western populations. While reduced mortality in the obesity range does not confirm metabolic health, it underscores the need to re‐evaluate BMI thresholds for Asians with T2D, as lower BMIs, particularly in the underweight range, were linked to significantly higher mortality risks. Even in a large pooled analysis of 20 prospective studies including over 1 million East and South Asian adults, a BMI of 25 kg/m^2^ or higher was associated with lower all‐cause mortality compared to lower BMI categories, further supporting the need to reconsider BMI thresholds for Asian populations with diabetes [38].

This study has certain limitations. Individuals with obesity may exhibit diverse phenotypes, including sarcopenic obesity and metabolically healthy obesity. In contrast, the underweight group likely comprises lean, fit individuals and cachexic older adults. These subgroups could not be segregated in the present study. Nevertheless, attempts were made to adjust for social history and underlying chronic diseases to address this heterogeneity. Diabetes severity was approximated using fasting glucose, medication burden and insulin use; however, inclusion of HbA1c, hypoglycemia history, time in range or complication status would have enabled a more comprehensive evaluation. Reverse causation is another potential limitation; chronic disease and frailty may lead to weight loss and increased mortality risk [39]. To reduce potential reverse causation, we performed sensitivity analyses excluding deaths within the first 1 and 2 years of follow‐up; the associations between underweight and all‐cause and cause‐specific mortality remained generally consistent and of comparable magnitude. Lastly, we relied on baseline BMI, without incorporating longitudinal changes in weight during follow‐up.

Nevertheless, the present study benefits from its large, nationally representative cohort of over 2.5 million adults with T2D, drawn from the Korean NHIS database. The inclusion of comprehensive medical and demographic data facilitated robust analyses and generalizability of findings to the South Korean population with T2D. Our findings not only align with previous studies but also provide novel insights into the risks faced by the understudied group of underweight individuals with T2D. We uniquely stratified underweight into mild, moderate and severe categories, revealing a stepwise increase in mortality risk with greater underweight severity. Notably, individuals with severe underweight had a higher risk of all‐cause mortality than those with class III obesity. Furthermore, we extended our analysis to cause‐specific outcomes and showed that the adverse impact of underweight status was consistent across diabetes‐related, cardiovascular and cerebrovascular mortality. We also applied comprehensive covariate adjustment, competing‐risk analyses and extensive subgroup analyses to address potential confounding and enhance the validity of our findings.

In conclusion, in this nationwide, retrospective cohort of adults with type 2 diabetes, underweight status was associated with higher all‐cause and cause‐specific mortality, with risks increasing from mild to severe underweight and exceeding those observed in class III obesity (Graphical Abstract). These observational findings support prioritizing the identification and clinical evaluation of underweight in diabetes care alongside obesity management. Future studies are warranted to clarify underlying mechanisms and to test whether targeted interventions (e.g., addressing malnutrition, sarcopenia or unintentional weight loss) can reduce risk in this vulnerable subgroup.

## Ethics Statement

This manuscript complies with the ethical guidelines for authorship and publication established by the *Journal of Cachexia, Sarcopenia and Muscle* (JCSM). All authors have made substantial and independent contributions to the work, have approved the final manuscript, and agree to its submission and potential publication.

## Conflicts of Interest

The authors declare no conflicts of interest.

## Supporting information


**Figure S1:** Cohort derivation flowchart.
**Table S1:** Missingness for critical covariates used in multivariable adjustment.
**Table S2:** Detailed characteristics of the study population across BMI ranges.
**Figure S2:** Cumulative incidence of mortality in underweight (BMI < 18.5 kg/m2) and non‐underweight (BMI ≥ 18.5 kg/m2) individuals with type 2 diabetes.
**Table S3:** Subgroup analysis of all‐cause mortality across BMI categories.
**Table S4:** Competing risk analysis for all‐cause and cause‐specific mortality across BMI categories.
**Table S5:** Lag‐time sensitivity analysis excluding deaths within the first year of follow‐up.
**Table S6:** Lag‐time sensitivity analysis excluding deaths within the first 2 years of follow‐up.

## Data Availability

The data used in this study was obtained from the Korean NHIS database. Access to these data is available upon reasonable request and approval from the Korean NHIS. Due to privacy regulations and data‐sharing policies, the data cannot be made publicly available.
