# Supplementary material for: Underweight and Mortality in Type 2 Diabetes: A Nationwide Retrospective Cohort Study
Source: J Cachexia Sarcopenia Muscle. 2025 Dec 5;16(6):e70145. doi: 10.1002/jcsm.70145 (PMC12680783; doi:10.1002/jcsm.70145)
Supplement: Supplementary file 1 — Figure S1: Cohort derivation flowchart. Table S1: Missingness for critical covariates used in multivariable adjustment. Table S2: Detailed characteristics of the study population across BMI ranges. Figure S2: Cumulative incidence of mortality in underweight (BMI < 18.5 kg/m2) and non‐underweight (BMI ≥ 18.5 kg/m2) individuals with type 2 diabetes. Table S3: Subgroup analysis of all‐cause mortality across BMI categories. Table S4: Competing risk analysis for all‐cause and cause‐specific mortality across BMI categories. Table S5: Lag‐time sensitivity analysis excluding deaths within the first year of follow‐up. Table S6: Lag‐time sensitivity analysis excluding deaths within the first 2 years of follow‐up. [file JCSM-16-e70145-s001.docx]

**Supplementary Material**

**Contents**

**Supplementary Method: Anthropometric measurement and baseline characteristics**2

**Supplementary Figure 1. Cohort derivation flowchart**4

**Supplementary Table 1. Missingness for critical covariates used in multivariable adjustment** 4

**Supplementary Table 2. Detailed characteristics of the study population across BMI ranges**5

**Supplementary Figure 2. Cumulative incidence of mortality in underweight (BMI <18.5 kg/m²) and non-underweight (BMI ≥18.5 kg/m²) individuals with type 2 diabetes**8

**Supplementary Table 3. Subgroup analysis of all-cause mortality across BMI categories**9

**Supplementary Table 4. Competing risk analysis for all-cause and cause-specific mortality across BMI categories**11

**Supplementary Table 5. Lag-time sensitivity analysis excluding deaths within the first year of follow-up**12

**Supplementary Table 6. Lag-time sensitivity analysis excluding deaths within the first two years of follow-up**13

**Supplementary Method: Anthropometric Measurement and Baseline Characteristics**

Trained medical staff measured anthropometric indices using standardized protocols. Height (cm) and body weight (kg) were assessed using calibrated electronic scales, and body mass index was calculated as weight in kilograms divided by the square of height in meters (kg/m²). Waist circumference was measured at the midpoint between the lower margin of the rib cage and the iliac crest.

Blood pressure was measured using a sphygmomanometer after participants had rested in a seated position for at least 5 minutes. Blood samples were collected after a minimum 8-hour fast to assess fasting plasma glucose, lipid profiles, and liver enzyme levels.

The participants were classified as follows according to smoking status: current smokers and ex-smokers or never smokers. Alcohol consumption was categorized as follows based on daily alcohol intake: no, moderate, and heavy (≥30 g/day and ≥20 g/day for males and females, respectively, regardless of the type of alcohol) alcohol consumption (1). Self-reported questionnaires completed during the health examinations were used to assess physical activity. Regular physical activity was defined as performing vigorous physical activity for at least 20 minutes per session ≥3 times per week or performing moderate physical activity for at least 30 minutes per session ≥5 times per week (2, 3). Participants with household income in the lowest 25th percentile were categorized as low-income.

Hypertension was defined as systolic blood pressure of ≥140 mmHg, diastolic blood pressure of ≥90 mmHg, or a history of receiving antihypertensive medication documented under ICD-10-CM codes of I10–I13 and I15. Dyslipidemia was defined as total cholesterol levels of ≥240 mg/dL or a history of receiving medication for dyslipidemia categorized under the ICD-10-CM code of E78. Chronic kidney disease (CKD) was defined as an estimated glomerular filtration rate (eGFR) of <60 mL/min/1.73 m², calculated using the Modification of Diet in Renal Disease (MDRD) equation. Central obesity was defined based on waist circumference, with thresholds of ≥90 cm for males and ≥85 cm for females, in accordance with the criteria recommended for the Korean population. Information regarding the duration of diabetes, insulin use, and the number of oral antidiabetic medications prescribed was obtained from medication claim records and prescription data under the ICD-10 codes of E11–E14.

The Charlson Comorbidity Index (CCI) was calculated using ICD-10 diagnostic codes obtained from claims data during the year prior to the index date. Each comorbidity was assigned a predefined weight (ranging from 1 to 6) according to the original CCI algorithm. Conditions included myocardial infarction, congestive heart failure, peripheral vascular disease, cerebrovascular disease, dementia, chronic pulmonary disease, rheumatologic disease, peptic ulcer disease, liver disease (mild, moderate, or severe), diabetes with or without complications, hemiplegia or paraplegia, renal disease, malignancy, metastatic solid tumor, and HIV/AIDS. The total CCI score for each participant was derived by summing the weights of all comorbid conditions identified.

**References for Supplementary Methods**

1. Kim YY, Kang HJ, Ha S, Park JH**.** Interactions of Behavioral Changes in Smoking, High-risk Drinking, and Weight Gain in a Population of 7.2 Million in Korea. J Prev Med Public Health. 2019;52(4):234-41.

2. Park KY, Huh Y, Nam GE, Han K, Jung JH, Cho YJ, et al. Changes in physical activity and all-cause mortality among individuals with dementia: a cohort study using the National Health Insurance Service Database in Korea. Br J Sports Med. 2024;58(21):1258-66.

3. Organization WH**.** WHO guidelines on physical activity and sedentary behaviour: World Health Organization; 2020.

4. Rhee EJ, Cho JH, Kwon H, Park SE, Jung JH, Han KD, Park YG, Kim YH, Lee WY. Relation between Baseline Height and New Diabetes Development: A Nationwide Population-Based Study. Diabetes Metab J. 2019 Dec;43(6):794-803.

5. Sundararajan V, Henderson T, Perry C, Muggivan A, Quan H, Ghali WA. New ICD-10 version of the Charlson comorbidity index predicted in-hospital mortality. J Clin Epidemiol. 2004 Dec;57(12):1288-94.

**Supplementary Figure 1. Cohort derivation flowchart**


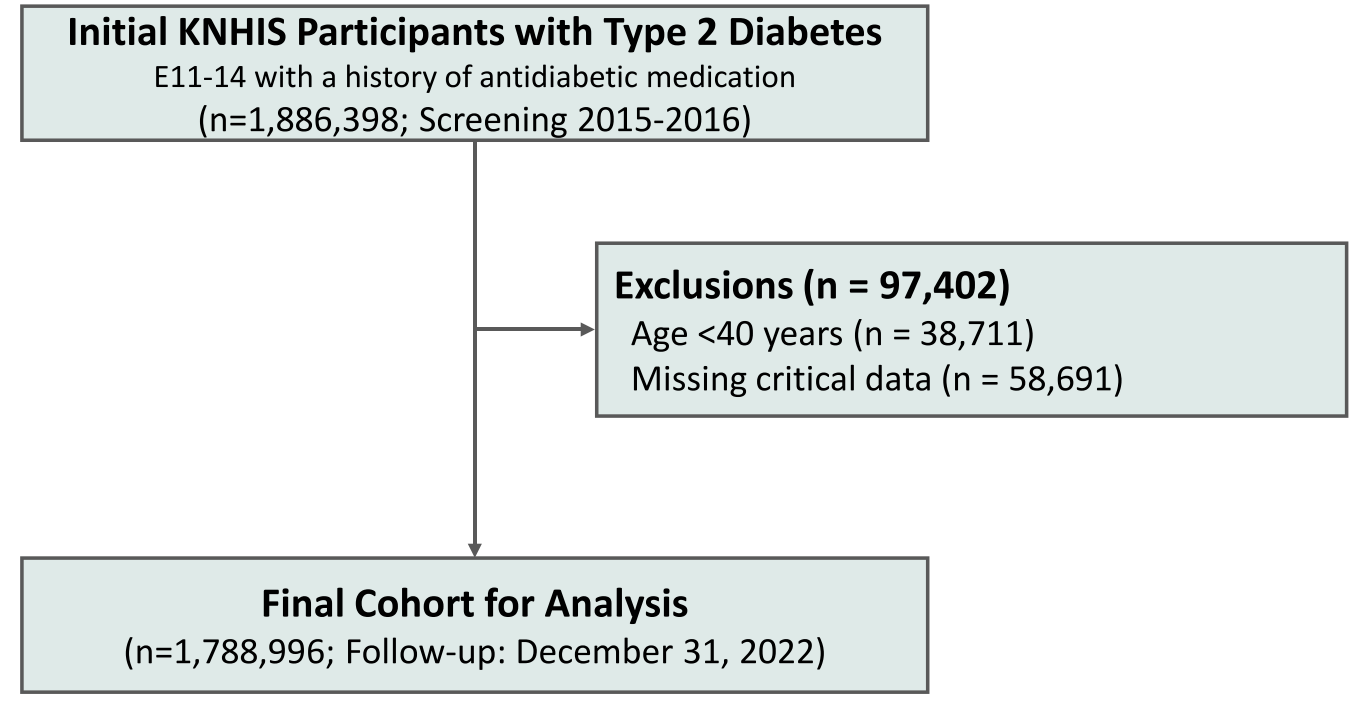


KNHIS, Korean National Health Insurance Service.

**Supplementary Table 1. Missingness for critical covariates used in multivariable adjustment**

|  |  |  |
| --- | --- | --- |
| **Variable** | **Missing number** | **Missing rate (%)** |
| Body mass index (BMI) | 1,421 | 0.075 |
| Anthropometric measures | 1,895 | 0.100 |
| Blood pressure | 388 | 0.021 |
| *Health behaviors* |  |  |
| Smoking history | 597 | 0.032 |
| Alcohol consumption | 2,276 | 0.121 |
| Regular exercise | 2,157 | 0.114 |
| Socioeconomic status (income) | 32,717 | 1.734 |
| *Laboratory measures* |  |  |
| Glucose | 222 | 0.012 |
| Cholesterol | 13,891 | 0.736 |
| Creatinine | 237 | 0.013 |
| Urine protein | 17,629 | 0.935 |
| Alanine aminotransferase (ALT) | 359 | 0.019 |
| Hemoglobin | 256 | 0.014 |
|  |  |  |

Values are the number (%) of the eligible pool prior to exclusions (N=1,886,398). Categories are grouped as anthropometrics, health behaviors/socioeconomic measures, and laboratory measures. Cholesterol variables include total cholesterol, high-density lipoprotein-cholesterol, low-density lipoprotein-cholesterol, and triglycerides. Blood pressure includes systolic and diastolic blood pressure measurements. Note that participants may be missing more than one variable.

**Supplementary Table 2. Detailed characteristics of the study population across BMI ranges**

|  | **BMI Group** | | | | | | | | *P* for trend |
| --- | --- | --- | --- | --- | --- | --- | --- | --- | --- |
|  | **Severely Underweight** | **Moderately Underweight** | **Mildly Underweight** | **Normal**  **Weight** | **Over-**  **weight** | **Class I**  **Obesity** | **Class II**  **Obesity** | **Class III Obesity** |  |
| kg/m^2^ | <16.0 | 16.0–16.9 | 17.0–18.4 | 18.5–22.9 | 23.0–24.9 | 25.0–29.9 | 30.0–34.9 | ≥35.0 |  |
| n (%) | 1,920  (0.1%) | 3,866  (0.2%) | 18,554  (1.0%) | 437,385  (24.4%) | 446,154  (24.9%) | 733,354  (41.0%) | 129,823  (7.3%) | 17,940  (1.0%) |  |
| BMI (kg/m^2^) | 15.1 ± 0.8 | 16.5 ± 0.3 | 17.9 ± 0.4 | 21.4 ± 1.1 | 24.0 ± 0.6 | 26.9 ± 1.3 | 31.6 ± 1.3 | 37.4 ± 2.6 | <.001 |
| Height (cm) | 159.2 ± 9.4 | 159.5 ± 9.2 | 160.1 ± 9.1 | 161.1 ± 8.9 | 161.9 ± 9.0 | 162.0 ± 9.3 | 161.1 ± 9.8 | 159.6 ± 10.3 | <.001 |
| Weight (kg) | 38.4 ± 5.0 | 42.2 ± 4.9 | 45.9 ± 5.3 | 55.8 ± 7.0 | 63.1 ± 7.2 | 70.9 ± 8.8 | 82.4 ± 10.5 | 95.6 ± 13.6 | <.001 |
| Waist circumference | 66.3 ± 7.5 | 68.0 ± 6.5 | 70.4 ± 5.9 | 78.3 ± 5.9 | 83.9 ± 5.4 | 89.8 ± 6.1 | 98.6 ± 6.9 | 108.5 ± 8.7 | <.001 |
| Central obesity | 29 (1.51) | 38 (0.98) | 195 (1.05) | 22,965 (5.25) | 100,294 (22.5) | 477,446 (65.1) | 125,774 (96.9) | 17,754 (99.0) | <.001 |
| Age (years) | 68.5 ± 12.8 | 67.0 ± 12.0 | 65.6 ± 11.6 | 64.1 ± 10.3 | 63.1 ± 10.1 | 61.8 ± 10.3 | 59.0 ± 10.8 | 56.1 ± 10.7 | <.001 |
| ≥65 | 1,156 (60.2) | 2,184 (56.5) | 9,659 (52.1) | 204,798 (46.8) | 195,339 (43.8) | 287,997 (39.3) | 39,914 (30.7) | 4,070 (22.7) | <.001 |
| Male (n, %) | 971 (50.6) | 2,006 (51.9) | 9,639 (52.0) | 238,098 (54.4) | 265,223 (59.5) | 427,432 (58.3) | 61,295 (47.2) | 6,306 (35.2) | <.001 |
| SBP (mmHg) | 119 ± 17 | 120 ± 17 | 122 ± 17 | 125 ± 15 | 128 ± 15 | 130 ± 15 | 132 ± 15 | 134 ± 15 | <.001 |
| DBP (mmHg) | 73 ± 11 | 73 ± 10 | 73 ± 10 | 75 ± 10 | 77 ± 9 | 78 ± 9 | 80 ± 10 | 82 ± 10 | <.001 |
| FPG (mg/dL) | 149 ± 83 | 148 ± 76 | 147 ± 69 | 142 ± 52 | 141 ± 46 | 141 ± 45 | 143 ± 45 | 144 ± 47 | <.001 |
| Cholesterol (mg/dL) | 171 ± 43 | 171 ± 41 | 172 ± 40 | 175 ± 41 | 176 ± 41 | 177 ± 41 | 179 ± 41 | 180 ± 41 | <.001 |
| Triglyceride* (mg/dL) | 96 (94, 98) | 93 (92, 95) | 94 (94, 95) | 113 (113, 113) | 128 (128, 129) | 141 (141, 141) | 150 (149, 150) | 148 (147, 149) | <.001 |
| HDL-C (mg/dL) | 58 ± 19 | 58 ± 22 | 57 ± 17 | 52 ± 15 | 50 ± 14 | 49 ± 13 | 49 ± 13 | 50 ± 13 | <.001 |
| LDL-C (mg/dL) | 92 ± 36 | 92 ± 35 | 94 ± 35 | 96 ± 36 | 97 ± 37 | 96 ± 37 | 97 ± 37 | 98 ± 36 | <.001 |
| eGFR | 80.4 ± 22.7 | 80.8 ± 22.6 | 81.6 ± 21.5 | 81.5 ± 20.2 | 81.2 ± 19.8 | 81.4 ± 19.9 | 83.1 ± 20.8 | 85.5 ± 21.0 | <.001 |
| *Social history* |  |  |  |  |  |  |  |  |  |
| Smoking (n, %) |  |  |  |  |  |  |  |  | - |
| Never smoker | 1,191 (62.0) | 2,336 (60.4) | 11,165 (60.2) | 261,549 (59.8) | 254,348 (57.0) | 420,136 (57.3) | 82,105 (63.2) | 12,617 (70.3) |  |
| Ex-smoker | 243 (12.7) | 563 (14.6) | 2,884 (15.5) | 86,705 (19.8) | 105,928 (23.7) | 179,474 (24.5) | 25,435 (19.6) | 2,538 (14.2) |  |
| Current smoker | 486 (25.3) | 967 (25.0) | 4,505 (24.3) | 89,131 (20.4) | 85,878 (19.3) | 133,744 (18.2) | 22,283 (17.2) | 2,785 (15.5) |  |
| Alcohol (n, %) |  |  |  |  |  |  |  |  | - |
| None | 1,524 (79.4) | 2,997 (77.5) | 13,718 (73.9) | 296,461 (67.8) | 282,633 (63.4) | 454,516 (62.0) | 85,395 (65.8) | 12,925 (72.1) |  |
| Mild | 296 (15.4) | 681 (17.6) | 3,738 (20.2) | 113,846 (26.0) | 131,787 (29.5) | 219,284 (29.9) | 34,295 (26.4) | 3,930 (21.9) |  |
| Heavy | 100 (5.21) | 188 (4.86) | 1,098 (5.92) | 27,078 (6.19) | 31,734 (7.11) | 59,554 (8.12) | 10,133 (7.81) | 1,085 (6.05) |  |
| Exercise (n, %) | 197 (10.3) | 604 (15.6) | 3,475 (18.7) | 102,399 (23.4) | 106,284 (23.8) | 159,419 (21.7) | 23,101 (17.8) | 2,629 (14.7) | <.001 |
| Low income (n, %) | 518 (27.0) | 1,033 (26.7) | 4,739 (25.5) | 98,302 (22.5) | 95,118 (21.3) | 158,420 (21.6) | 30,767 (23.7) | 4,758 (26.5) | 0.455 |
| *Medical history* |  |  |  |  |  |  |  |  |  |
| Hypertension (n, %) | 901 (46.9) | 1,821 (47.1) | 8,608 (46.4) | 237,706 (54.4) | 278,117 (62.3) | 519,189 (70.8) | 103,644 (79.8) | 15,377 (85.7) | <.001 |
| Dyslipidemia (n, %) | 758 (39.5) | 1,596 (41.3) | 8,586 (46.3) | 262,977 (60.1) | 292,171 (65.5) | 506,050 (69.0) | 94,113 (72.5) | 13,070 (72.9) | <.001 |
| CKD (n, %) | 353 (18.4) | 675 (17.5) | 2,908 (15.7) | 60,009 (13.7) | 60,022 (13.5) | 99,123 (13.5) | 16,713 (12.9) | 2,116 (11.8) | <.001 |
| Diabetes duration (years) |  |  |  |  |  |  |  |  | - |
| New onset | 871 (33.9) | 1,765 (32.4) | 8,113 (31.0) | 154,472 (26.4) | 169,605 (28.0) | 333,665 (31.9) | 74,929 (36.9) | 14,019 (42.3) |  |
| <5 years | 488 (19.0) | 983 (18.1) | 4,689 (17.9) | 119,388 (20.46) | 138,282 (22.8) | 263,351 (25.2) | 57,211 (28.2) | 9,853 (29.7) |  |
| <10 years | 497 (19.4) | 1,013 (18.6) | 4,932 (18.9) | 122,550 (20.9) | 130,626 (21.6) | 219,477 (21.0) | 39,124 (19.3) | 5,448 (16.5) |  |
| ≥10 years | 712 (27.7) | 1,683 (30.9) | 8,413 (32.2) | 189,891 (32.4) | 167,685 (27.7) | 228,838 (21.9) | 31,598 (15.6) | 3,805 (11.5) |  |
| ≥3 OADs | 475 (18.5) | 1,162 (21.3) | 5,847 (22.4) | 141,022 (24.1) | 143,795 (23.7) | 246,332 (23.6) | 49,246 (24.3) | 8,043 (24.3) | .459 |
| Insulin use (n, %) | 378 (14.7) | 827 (15.2) | 3,727 (14.3) | 60,021 (10.2) | 50,883 (8.4) | 80,561 (7.7) | 15,128 (7.5) | 2,399 (7.2) | <.001 |
| **CCI** | 4.05 ± 2.45 | 4.01 ± 2.44 | 3.83 ± 2.38 | 3.54 ± 2.21 | 3.43 ± 2.14 | 3.41 ± 2.12 | 3.45 ± 2.12 | 3.48 ± 2.14 | <.001 |
| MI (n, %) | 43 (2.24) | 72 (1.86) | 321 (1.73) | 6,788 (1.55) | 7,032 (1.58) | 11,705 (1.60) | 1,957 (1.51) | 246 (1.37) | 0.318 |
| CHF (n, %) | 120 (6.25) | 244 (6.31) | 919 (4.95) | 17,416 (3.98) | 17,534 (3.93) | 30,699 (4.19) | 6,482 (4.99) | 1,104 (6.15) | <.001 |
| PVD (n, %) | 407 (21.2) | 861 (22.3) | 4,121 (22.2) | 92,753 (21.2) | 93,221 (20.9) | 152,910 (20.9) | 27,253 (21.0) | 3,756 (20.9) | <.001 |
| Cerebrovascular disease (n, %) | 403 (21.0) | 750 (19.4) | 3,021 (16.3) | 61,933 (14.2) | 60,436 (13.6) | 95,649 (13.0) | 15,478 (11.9) | 1,878 (10.5) | <.001 |
| Dementia (n, %) | 344 (17.9) | 431 (11.2) | 1,407 (7.58) | 16,727 (3.82) | 12,347 (2.77) | 17,337 (2.36) | 2,613 (2.01) | 280 (1.56) | <.001 |
| Chronic pulmonary disease (n, %) | 699 (36.4) | 1,364 (35.3) | 5,841 (31.5) | 128,274 (29.3) | 130,209 (29.2) | 219,139 (29.9) | 41,296 (31.8) | 6,118 (34.1) | <.001 |
| Rheumatologic disease | 117 (6.09) | 208 (5.38) | 1,015 (5.47) | 20,824 (4.76) | 19,899 (4.46) | 32,825 (4.48) | 6,198 (4.77) | 895 (4.99) | <.001 |
| Peptic ulcer disease | 646 (33.7) | 1,240 (32.1) | 5,739 (30.9) | 127,136 (29.1) | 126,688 (28.4) | 206,169 (28.1) | 36,174 (27.9) | 4,846 (27.0) | <.001 |
| Mild liver disease | 760 (39.6) | 1,562 (40.4) | 7,267 (39.2) | 163,243 (37.3) | 169,725 (38.0) | 296,131 (40.4) | 57,437 (44.2) | 8,368 (46.6) | <.001 |
| Moderate/severe liver disease (n, %) | 116 (6.04) | 247 (6.39) | 1,112 (5.99) | 22,812 (5.22) | 22,210 (4.98) | 37,220 (5.08) | 6,831 (5.26) | 993 (5.54) | 0.022 |
| Hemiplegia or paraplegia (n, %) | 1,585 (82.6) | 3,224 (83.4) | 15,269 (82.3) | 361,167 (82.6) | 367,219 (82.3) | 600,574 (81.9) | 105,842 (81.5) | 14,593 (81.3) | <.001 |
| Any malignancy (n, %) | 47 (2.45) | 52 (1.35) | 232 (1.25) | 3,107 (0.71) | 2,686 (0.6) | 3,842 (0.52) | 582 (0.45) | 89 (0.50) | <.001 |
| Metastatic solid tumor | 353 (18.4) | 764 (19.8) | 3,515 (18.9) | 73,392 (16.8) | 70,547 (15.8) | 112,967 (15.4) | 19,752 (15.2) | 2,701 (15.1) | <.001 |
| AIDS/HIV (n, %) | 19 (0.99) | 39 (1.01) | 170 (0.92) | 2,687 (0.61) | 2,300 (0.52) | 3,703 (0.50) | 728 (0.56) | 98 (0.55) | <.001 |

Data are presented as mean ± standard deviation (SD) for continuous variables or n (%) for categorical variables. ***Triglycerides are presented as geometric mean (95% confidence interval) after log transformation due to right-skewed distribution.** BMI, body mass index; CKD, chronic kidney disease; CCI, Charlson Comorbidity Index; MI, myocardial infarction; CHF, congestive heart failure; PVD, peripheral vascular disease; HDL-C, high-density lipoprotein cholesterol; LDL-C, low-density lipoprotein cholesterol; SBP, systolic blood pressure; DBP, diastolic blood pressure; FPG, fasting plasma glucose; OADs, oral antidiabetic drugs; CVD, cardiovascular disease. *P* value indicates a test for trend.

**Supplementary Figure 2. Cumulative incidence of mortality in underweight (BMI <18.5 kg/m²) and non-underweight (BMI ≥18.5 kg/m²) individuals with type 2 diabetes.**


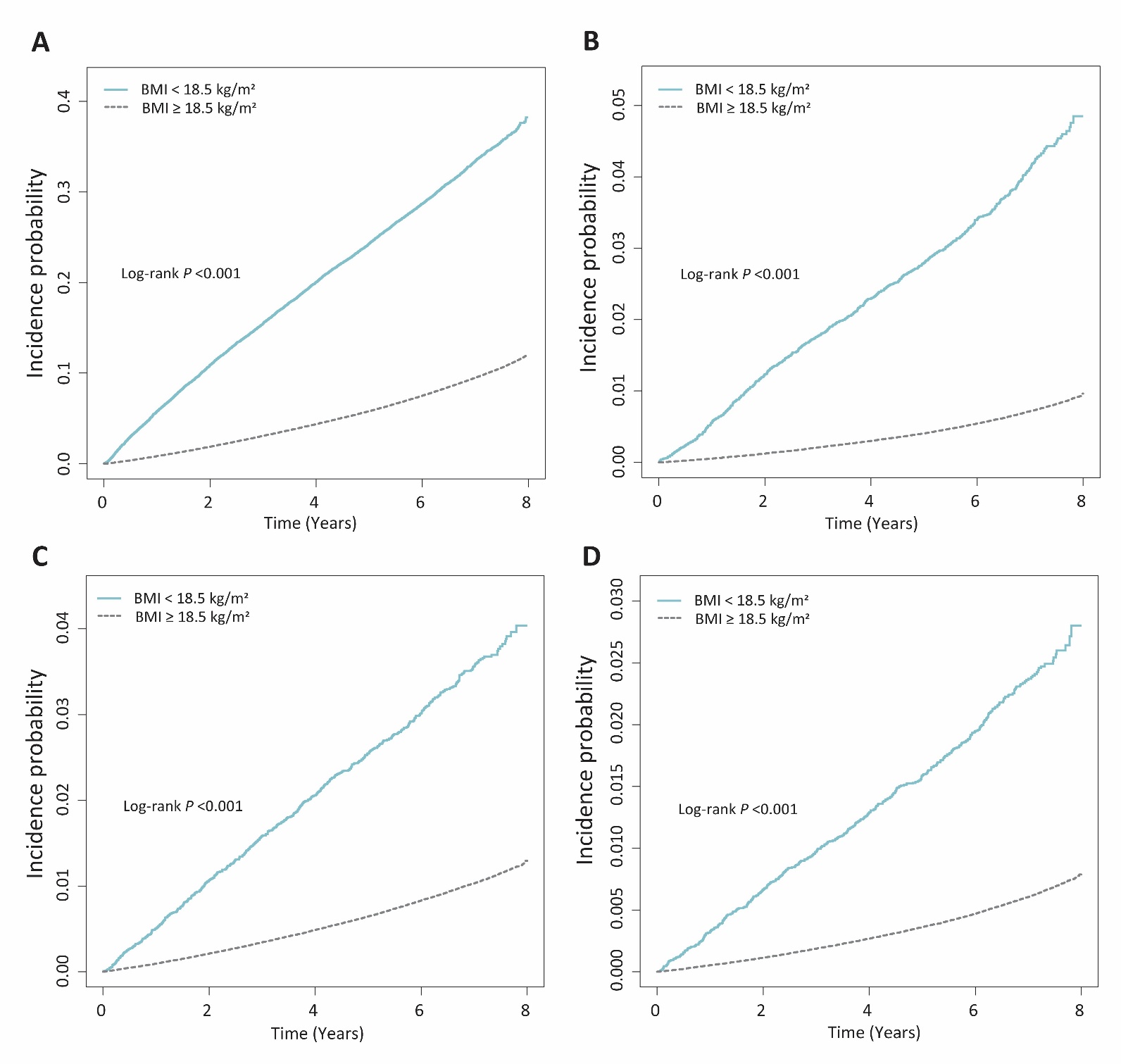


(A) All-cause mortality, (B) Diabetes-related mortality, (C) Cardiovascular disease-related mortality, and (D) Cerebrovascular disease-related mortality.

BMI, body mass index.

**Supplementary Table 3. Subgroup analysis of all-cause mortality across BMI categories**

| **Subgroup** | **Criteria** | **BMI Group** | **N** | **Event** | **Duration** | **IR per 1,000** | **Fully Adjusted Model**  **HR (95% CI)** | ***P* for interaction** |
| --- | --- | --- | --- | --- | --- | --- | --- | --- |
| Age | 40≤Age<65 | <16.0 | 764 | 278 | 4,240 | 66 | 6.190 (5.500–6.967) | <.0001 |
| (years) |  | 16.0–16.9 | 1,682 | 406 | 10,250 | 40 | 4.067 (3.688–4.486) |  |
|  |  | 17.0–18.4 | 8,895 | 1,401 | 57,051 | 25 | 2.789 (2.644–2.943) |  |
|  |  | ≥18.5 | 1,032,538 | 42,150 | 7,055,902 | 6 | 1 (ref.) |  |
|  | Age ≥65 | <16.0 | 1,156 | 771 | 4,827 | 160 | 3.354 (3.124–3.601) |  |
|  |  | 16.0–16.9 | 2,184 | 1,189 | 10,817 | 110 | 2.417 (2.282–2.560) |  |
|  |  | 17.0–18.4 | 9,659 | 4,059 | 53,262 | 76 | 1.849 (1.791–1.908) |  |
|  |  | ≥18.5 | 732,118 | 125,802 | 4,766,617 | 26 | 1 (ref.) |  |
| Sex | Male | <16.0 | 971 | 597 | 4,282 | 139 | 3.801 (3.507–4.121) | <.0001 |
|  |  | 16.0–16.9 | 2,006 | 973 | 10,308 | 94 | 2.620 (2.459–2.791) |  |
|  |  | 17.0–18.4 | 9,639 | 3,458 | 55,033 | 63 | 1.951 (1.885–2.019) |  |
|  |  | ≥18.5 | 998,354 | 108,013 | 6,641,267 | 16 | 1 (ref.) |  |
|  | Female | <16.0 | 949 | 452 | 4,785 | 94 | 3.970 (3.619–4.356) |  |
|  |  | 16.0–16.9 | 1,860 | 622 | 10,758 | 58 | 2.888 (2.668–3.126) |  |
|  |  | 17.0–18.4 | 8,915 | 2002 | 55,280 | 36 | 2.204 (2.107–2.305) |  |
|  |  | ≥18.5 | 766,302 | 59,939 | 5,181,252 | 12 | 1 (ref.) |  |
| Chronic kidney disease | (-) | <16.0 | 1,567 | 813 | 7,628 | 107 | 4.455 (4.157–4.775) | <.0001 |
|  |  | 16.0–16.9 | 3,191 | 1,150 | 18,074 | 64 | 2.945 (2.778–3.122) |  |
|  |  | 17.0–18.4 | 15,646 | 3,986 | 95,453 | 42 | 2.225 (2.155–2.297) |  |
|  |  | ≥18.5 | 1,526,673 | 112,569 | 10,323,466 | 11 | 1 (ref.) |  |
|  | (+) | <16.0 | 353 | 236 | 1,439 | 164 | 3.106 (2.733–3.529) |  |
|  |  | 16.0–16.9 | 675 | 445 | 2,992 | 149 | 2.541 (2.314–2.790) |  |
|  |  | 17.0–18.4 | 2,908 | 1,474 | 14,861 | 99 | 1.817 (1.725–1.914) |  |
|  |  | ≥18.5 | 237,983 | 55,383 | 1,499,053 | 37 | 1 (ref.) |  |
| Previous CVD history | (-) | <16.0 | 1,493 | 743 | 7,428 | 100 | 4.059 (3.775–4.364) | 0.0005 |
|  |  | 16.0–16.9 | 3,111 | 1,129 | 17,717 | 64 | 2.759 (2.601–2.926) |  |
|  |  | 17.0–18.4 | 15,509 | 4,015 | 94,422 | 43 | 2.105 (2.039–2.173) |  |
|  |  | ≥18.5 | 1,530,246 | 123,488 | 10,315,330 | 12 | 1 (ref.) |  |
|  | (+) | <16.0 | 427 | 306 | 1,638 | 187 | 3.445 (3.078–3.855) |  |
|  |  | 16.0–16.9 | 755 | 466 | 3,349 | 139 | 2.641 (2.410–2.894) |  |
|  |  | 17.0–18.4 | 3,045 | 1,445 | 15,891 | 91 | 1.892 (1.795–1.995) |  |
|  |  | ≥18.5 | 234,410 | 44,464 | 1,507,189 | 30 | 1 (ref.) |  |
| Smoking status | Never/  Ex-smoker | <16.0 | 1,434 | 793 | 6,719 | 118 | 3.980 (3.711–4.269) | 0.3766 |
|  |  | 16.0–16.9 | 2,899 | 1,173 | 15,854 | 74 | 2.761 (2.606–2.925) |  |
|  |  | 17.0–18.4 | 14,049 | 4,043 | 83,865 | 48 | 2.040 (1.977–2.106) |  |
|  |  | ≥18.5 | 1,430,835 | 136,365 | 9,599,136 | 14 | 1 (ref.) |  |
|  | Current smoker | <16.0 | 486 | 256 | 2,348 | 109 | 3.585 (3.170–4.055) |  |
|  |  | 16.0–16.9 | 967 | 422 | 5,213 | 81 | 2.607 (2.368–2.870) |  |
|  |  | 17.0–18.4 | 4505 | 1,417 | 26,449 | 54 | 2.027 (1.921–2.138) |  |
|  |  | ≥18.5 | 333,821 | 31,587 | 2,223,384 | 14 | 1 (ref.) |  |

BMI categories are defined as follows: Severe underweight, <16.0 kg/m²; Moderate underweight, 16.0–16.9 kg/m²; Mild underweight, 17.0–18.4 kg/m²; and Normal or higher, ≥18.5 kg/m². The model is adjusted for age, sex, income level, smoking status, alcohol consumption, physical activity, Charlson Comorbidity Index, fasting glucose, use of ≥3 oral antidiabetic medications or insulin, duration of diabetes, hemoglobin, and alanine aminotransferase. Hazard ratios (HRs) are presented with 95% confidence intervals (CIs).

**Supplementary Table 4. Competing risk analysis for all-cause and cause-specific mortality across BMI categories**

| **Outcome** | **BMI groups (kg/m^2^)** | **N** | **Event** | **Duration** | **Model 1** | **Model 2** | **Model 3** | **Model 4** |
| --- | --- | --- | --- | --- | --- | --- | --- | --- |
| All-cause mortality | <16.0 | 1,920 | 1,049 | 9,067 | 8.588 (8.043–9.171) | 4.998 (4.643–5.381) | 4.639 (4.305–5.000) | 3.843 (3.555–4.154) |
|  | 16.0–16.9 | 3,866 | 1,595 | 21,066 | 5.508 (5.233–5.796) | 3.438 (3.245–3.641) | 3.181 (2.999–3.373) | 2.705 (2.551–2.868) |
|  | 17.0–18.4 | 18,554 | 5,460 | 110,313 | 3.550 (3.454–3.649) | 2.494 (2.421–2.569) | 2.313 (2.244–2.384) | 2.032 (1.970–2.096) |
|  | ≥18.5 | 1,764,656 | 167,952 | 11,822,519 | 1 (ref.) | 1 (ref.) | 1 (ref.) | 1 (ref.) |
|  | *P* |  |  |  | <.0001 | <.0001 | <.0001 | <.0001 |
| Diabetes mortality | <16.0 | 1,920 | 125 | 9,923 | 13.206 (11.048–15.785) | 6.961 (5.775–8.392) | 6.163 (5.091–7.460) | 4.569 (3.749–5.567) |
|  | 16.0–16.9 | 3,866 | 176 | 22,667 | 7.964 (6.858–9.249) | 4.446 (3.803–5.198) | 3.788 (3.222–4.453) | 2.952 (2.495–3.492) |
|  | 17.0–18.4 | 18,554 | 537 | 116,194 | 4.680 (4.292–5.103) | 3.068 (2.806–3.355) | 2.587 (2.361–2.835) | 2.115 (1.927–2.321) |
|  | ≥18.5 | 1,764,656 | 12,115 | 12,039,871 | 1 (ref.) | 1 (ref.) | 1 (ref.) | 1 (ref.) |
|  | *P* |  |  |  | <.0001 | <.0001 | <.0001 | <.0001 |
| Cardiovascular disease mortality | <16.0 | 1,920 | 80 | 9,923 | 5.742 (4.604–7.161) | 3.047 (2.433–3.816) | 2.968 (2.370–3.719) | 2.538 (2.022–3.186) |
|  | 16.0–16.9 | 3,866 | 127 | 22,667 | 3.938 (3.305–4.692) | 2.238 (1.872–2.674) | 2.160 (1.805–2.583) | 1.891 (1.580–2.263) |
|  | 17.0–18.4 | 18,554 | 521 | 116,194 | 3.125 (2.864–3.410) | 2.069 (1.893–2.260) | 1.983 (1.813–2.169) | 1.780 (1.626–1.948) |
|  | ≥18.5 | 1,764,656 | 17,495 | 12,039,871 | 1 (ref.) | 1 (ref.) | 1 (ref.) | 1 (ref.) |
|  | *P* |  |  |  | <.0001 | <.0001 | <.0001 | <.0001 |
| Cerebrovascular disease mortality | <16.0 | 1,920 | 58 | 9,923 | 7.167 (5.531–9.287) | 3.770 (2.902–4.897) | 3.501 (2.693–4.552) | 3.157 (2.426–4.108) |
|  | 16.0–16.9 | 3,866 | 81 | 22,667 | 4.303 (3.458–5.355) | 2.463 (1.976–3.069) | 2.272 (1.822–2.833) | 2.088 (1.673–2.604) |
|  | 17.0–18.4 | 18,554 | 339 | 116,194 | 3.479 (3.122–3.876) | 2.315 (2.074–2.584) | 2.139 (1.914–2.391) | 1.991 (1.781–2.227) |
|  | ≥18.5 | 1,764,656 | 10,281 | 12,039,871 | 1 (ref.) | 1 (ref.) | 1 (ref.) | 1 (ref.) |
|  | *P* |  |  |  | <.0001 | <.0001 | <.0001 | <.0001 |

Data are presented as hazard ratios (95% confidence intervals).

Model adjustments: Model 1, unadjusted; Model 2, age, sex, income level, smoking status, alcohol consumption, physical activity, and the Charlson Comorbidity Index; Model 3, Model 2 + fasting glucose, use of ≥3 oral antidiabetic medications or insulin, and duration of diabetes; Model 4, Model 3 + hemoglobin and alanine aminotransferase. BMI, body mass index.

**Supplementary Table 5. Lag-time sensitivity analysis excluding deaths within the first year of follow-up**

| **Outcome** | **BMI groups (kg/m^2^)** | **Model 1** | **Model 2** | **Model 3** | **Model 4** |
| --- | --- | --- | --- | --- | --- |
| All-cause mortality | <16.0 | 7.281 (6.785–7.813) | 4.358 (4.061–4.677) | 4.094 (3.815–4.395) | 3.459 (3.223–3.713) |
|  | 16.0–16.9 | 5.027 (4.759–5.309) | 3.199 (3.029–3.379) | 3.000 (2.840–3.169) | 2.593 (2.454–2.739) |
|  | 17.0–18.4 | 3.353 (3.257–3.453) | 2.378 (2.309–2.448) | 2.229 (2.164–2.296) | 1.983 (1.925–2.043) |
|  | ≥18.5 | 1 (ref.) | 1 (ref.) | 1 (ref.) | 1 (ref.) |
|  | *P* | <.0001 | <.0001 | <.0001 | <.0001 |
| Diabetes mortality | <16.0 | 12.085 (9.854–14.822) | 6.818 (5.557–8.364) | 5.980 (4.871–7.340) | 4.511 (3.674–5.540) |
|  | 16.0–16.9 | 7.719 (6.549–9.098) | 4.624 (3.922–5.452) | 3.935 (3.334–4.644) | 3.118 (2.641–3.681) |
|  | 17.0–18.4 | 4.679 (4.266–5.131) | 3.194 (2.912–3.504) | 2.710 (2.467–2.976) | 2.243 (2.042–2.465) |
|  | ≥18.5 | 1 (ref.) | 1 (ref.) | 1 (ref.) | 1 (ref.) |
|  | *P* | <.0001 | <.0001 | <.0001 | <.0001 |
| Cardiovascular disease mortality | <16.0 | 5.399 (4.190–6.957) | 3.027 (2.349–3.902) | 2.956 (2.293–3.811) | 2.540 (1.970–3.275) |
|  | 16.0–16.9 | 3.776 (3.105–4.592) | 2.279 (1.873–2.772) | 2.217 (1.822–2.698) | 1.948 (1.600–2.371) |
|  | 17.0–18.4 | 3.119 (2.839–3.426) | 2.139 (1.947–2.351) | 2.061 (1.874–2.267) | 1.852 (1.683–2.037) |
|  | ≥18.5 | 1 (ref.) | 1 (ref.) | 1 (ref.) | 1 (ref.) |
|  | *P* | <.0001 | <.0001 | <.0001 | <.0001 |
| Cerebrovascular disease mortality | <16.0 | 6.939 (5.177–9.299) | 3.851 (2.873–5.164) | 3.606 (2.688–4.837) | 3.273 (2.439–4.391) |
|  | 16.0–16.9 | 4.399 (3.471–5.574) | 2.665 (2.103–3.379) | 2.490 (1.963–3.159) | 2.297 (1.811–2.915) |
|  | 17.0–18.4 | 3.408 (3.030–3.832) | 2.341 (2.081–2.634) | 2.183 (1.938–2.459) | 2.036 (1.807–2.294) |
|  | ≥18.5 | 1 (ref.) | 1 (ref.) | 1 (ref.) | 1 (ref.) |
|  | *P* | <.0001 | <.0001 | <.0001 | <.0001 |

Data are presented as hazard ratios (95% confidence intervals).

Model adjustments: Model 1, unadjusted; Model 2, age, sex, income level, smoking status, alcohol consumption, physical activity, and the Charlson Comorbidity Index; Model 3, Model 2 + fasting glucose, use of ≥3 oral antidiabetic medications or insulin, and duration of diabetes; Model 4, Model 3 + hemoglobin and alanine aminotransferase. BMI, body mass index.

**Supplementary Table 6. Lag-time sensitivity analysis excluding deaths within the first two years of follow-up**

| **Outcome** | **BMI groups (kg/m^2^)** | **Model 1** | **Model 2** | **Model 3** | **Model 4** |
| --- | --- | --- | --- | --- | --- |
| All-cause mortality | <16.0 | 6.752 (6.235–7.311) | 4.154 (3.836–4.499) | 3.933 (3.631–4.260) | 3.370 (3.111–3.650) |
|  | 16.0–16.9 | 4.729 (4.450–5.026) | 3.074 (2.892–3.267) | 2.911 (2.738–3.094) | 2.544 (2.393–2.705) |
|  | 17.0–18.4 | 3.178 (3.077–3.282) | 2.281 (2.209–2.356) | 2.155 (2.086–2.227) | 1.933 (1.871–1.997) |
|  | ≥18.5 | 1 (ref.) | 1 (ref.) | 1 (ref.) | 1 (ref.) |
|  | *P* | <.0001 | <.0001 | <.0001 | <.0001 |
| Diabetes mortality | <16.0 | 9.872 (7.736–12.598) | 5.756 (4.509–7.348) | 5.097 (3.991–6.511) | 3.885 (3.041–4.964) |
|  | 16.0–16.9 | 6.414 (5.287–7.781) | 3.954 (3.258–4.798) | 3.405 (2.804–4.136) | 2.720 (2.238–3.304) |
|  | 17.0–18.4 | 4.364 (3.942–4.832) | 3.033 (2.739–3.359) | 2.609 (2.352–2.893) | 2.167 (1.954–2.404) |
|  | ≥18.5 | 1 (ref.) | 1 (ref.) | 1 (ref.) | 1 (ref.) |
|  | *P* | <.0001 | <.0001 | <.0001 | <.0001 |
| Cardiovascular disease mortality | <16.0 | 4.968 (3.721–6.633) | 2.846 (2.130–3.802) | 2.788 (2.086–3.727) | 2.416 (1.807–3.230) |
|  | 16.0–16.9 | 3.434 (2.749–4.290) | 2.112 (1.691–2.640) | 2.069 (1.655–2.587) | 1.832 (1.465–2.291) |
|  | 17.0–18.4 | 2.909 (2.619–3.231) | 2.018 (1.816–2.242) | 1.956 (1.759–2.175) | 1.766 (1.587–1.964) |
|  | ≥18.5 | 1 (ref.) | 1 (ref.) | 1 (ref.) | 1 (ref.) |
|  | *P* | <.0001 | <.0001 | <.0001 | <.0001 |
| Cerebrovascular disease mortality | <16.0 | 5.789 (4.091–8.192) | 3.345 (2.364–4.733) | 3.153 (2.227–4.464) | 2.891 (2.042–4.094) |
|  | 16.0–16.9 | 4.053 (3.109–5.284) | 2.530 (1.940–3.299) | 2.391 (1.833–3.121) | 2.224 (1.704–2.903) |
|  | 17.0–18.4 | 3.228 (2.838–3.672) | 2.263 (1.989–2.576) | 2.133 (1.872–2.431) | 2.002 (1.756–2.281) |
|  | ≥18.5 | 1 (ref.) | 1 (ref.) | 1 (ref.) | 1 (ref.) |
|  | *P* | <.0001 | <.0001 | <.0001 | <.0001 |

Data are presented as hazard ratios (95% confidence intervals).

Model adjustments: Model 1, unadjusted; Model 2, age, sex, income level, smoking status, alcohol consumption, physical activity, and the Charlson Comorbidity Index; Model 3, Model 2 + fasting glucose, use of ≥3 oral antidiabetic medications or insulin, and duration of diabetes; Model 4, Model 3 + hemoglobin and alanine aminotransferase. BMI, body mass index.
